# Reduced 5-FU clearance in a patient with low DPD activity due to heterozygosity for a mutant allele of the DPYD gene

**DOI:** 10.1038/sj.bjc.6600208

**Published:** 2002-04-08

**Authors:** J G Maring, A B P van Kuilenburg, J Haasjes, H Piersma, H J M Groen, D R A Uges, A H Van Gennip, E G E De Vries

**Affiliations:** Department of Pharmacy, Diaconessen Hospital, Meppel and Bethesda Hospital, Hoogeveen, Hoogeveenseweg 38, 7943 KA Meppel, The Netherlands; Department of Clinical Chemistry, Academic Medical Center and Emma Children's Hospital, University of Amsterdam, Meibergdreef 9, 1105 AZ Amsterdam, The Netherlands; Department of Internal Medicine, Martini Hospital, Van Swietenlaan 4, 9728 NZ Groningen, The Netherlands; Department of Pulmonary Medicine, University Hospital, Hanzeplein 1, 9713 GZ Groningen, The Netherlands; Department of Pharmacy, University Hospital, Hanzeplein 1, 9713 GZ Groningen, The Netherlands; Department of Medical Oncology, University Hospital, Hanzeplein 1, 9713 GZ Groningen, The Netherlands

**Keywords:** DPD, 5-fluorouracil, pharmacokinetics, DPYD gene, mutation, pharmacogenetics

## Abstract

5-fluorouracil pharmacokinetics, dihydropyrimidine dehydrogenase-activity and DNA sequence analysis were compared between a patient with extreme 5-fluorouracil induced toxicity and six control patients with normal 5-fluorouracil related symptoms. Patients were treated for colorectal cancer and received chemotherapy consisting of leucovorin 20 mg m^−2^ plus 5-fluorouracil 425 mg m^−2^. Blood sampling was carried out on day 1 of the first cycle. The 5-fluorouracil area under the curve_0→3h_ in the index patient was 24.1 mg h l^−1^ compared to 9.8±3.6 (range 5.4–15.3) mg h l^−1^ in control patients. The 5-fluorouracil clearance was 520 ml min^−1^ *vs* 1293±302 (range 980–1780) ml min^−1^ in controls. The activity of dihydropyrimidine dehydrogenase in mononuclear cells was lower in the index patient (5.5 nmol mg h^−1^) compared to the six controls (10.3±1.6, range 8.0–11.7 nmol mg h^−1^). Sequence analysis of the dihydropyrimidine dehydrogenase gene revealed that the index patient was heterozygous for a IVS14+1G>A point mutation. Our results indicate that the inactivation of one dihydropyrimidine dehydrogenase allele can result in a strong reduction in 5-fluorouracil clearance, causing severe 5-fluorouracil induced toxicity.

*British Journal of Cancer* (2002) **86**, 1028–1033. DOI: 10.1038/sj/bjc/6600199 www.bjcancer.com

© 2002 Cancer Research UK

## 

Fluorouracil (5-FU) is widely used in chemotherapeutic regimens for the treatment of breast-, colorectal- and head- and neck cancer. The cytotoxic mechanism of 5-FU is complex, requiring intracellular bioconversion of 5-FU into cytotoxic nucleotides (see Figure 1

**Figure 1:**
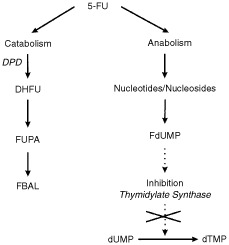
Metabolism of 5-FU. 5-Fluoro-2′-deoxyuridine-5′-monophosphate (FdUMP) is the cytotoxic product resulting from a multi-step 5-FU activation route. FdUMP inhibits the enzyme thymidylate synthase (TS), which leads to intracellular accumulation of deoxy-uridine-monophospate (dUMP) and depletion of deoxy-thymidine-monophosphate (dTMP). This causes arrest of DNA synthesis. The initial and rate-limiting enzyme in the catabolism of 5-FU is dihydropyrimidine dehydrogenase (DPD), catalysing the reduction of 5-FU into 5,6-dihydrofluorouracil (DHFU). Subsequently, DHFU is degraded into fluoro-β-ureidopropionic acid (FUPA) and fluoro-β-alanine (FBAL).

). Inhibition of thymidylate synthase by the metabolite 5-fluoro-2′-deoxyuridine-5′-monophosphate is thought to be the main mechanism of cytotoxicity (Pinedo and Peters, 1988) . The cytotoxicity is caused by only a small part of the administered 5-FU dose, as the majority of 5-FU is rapidly metabolised into inactive metabolites. The initial and rate-limiting enzyme in the catabolism of 5-FU is dihydropyrimidine dehydrogenase (DPD), catalysing the reduction of 5-FU into 5,6-dihydrofluorouracil (DHFU). Several groups have suggested a major role of DPD in the regulation of 5-FU metabolism and thus in the amount of 5-FU available for cytotoxicity (Harris *et al*, 1990; Fleming *et al*, 1992a; Lu *et al*, 1993; Etienne *et al*, 1994). Indeed, in patients with DPD enzyme deficiency, 5-FU chemotherapy is associated with severe, life-threatening toxicity (Van Kuilenburg *et al*, 2000a). Moreover, a markedly prolonged elimination half-life of 5-FU has been observed in a patient with complete deficiency of DPD enzyme activity (Diasio *et al*, 1988). Several mutations in the dihydropyrimidine dehydrogenase gene (DPYD), which encodes for the DPD enzyme have recently been identified (Van Kuilenburg *et al*, 2000a; Collie-Duguid *et al*, 2000). Furthermore, the frequency of DPD deficiency has been estimated to be as high as 2–3% (Etienne *et al*, 1994; Lu *et al*, 1995; Chazal *et al*, 1996). To date, a direct correlation between DPYD gene mutation and decreased 5-FU clearance has only been suggested but never been proven. In this study, we provide the first detailed analysis of 5-FU pharmacokinetics in a patient with low DPD-activity due to heterozygosity for a mutant allele of the gene encoding DPD.

## METHODS

### Chemicals

5-Fluorouracil was obtained from Sigma Chemical Co. (Zwijndrecht, The Netherlands). 5,6-Dihydro-5-fluorouracil was kindly provided by Roche Laboratories (Basel, Switzerland). AmpliTaq Taq polymerase and BigDye-Terminator-Cycle-Sequencing-Ready-Reaction kit were supplied by Perkin Elmer (San Jose, CA, USA). A Quaquik Gel Extraction kit was obtained from Qiagen (Hilden, Germany). Human heparinised plasma was obtained from the Red Cross Blood Bank (Groningen, The Netherlands). [4-^14^C]Thymine (1.85–2.22 GBq mmol^−1^) was obtained from Moravek Biochemicals (CA, USA) and Lymphoprep (spec.gravity 1.077 g ml^−1^, 280 mOsm) was from Nycomed Pharma AS (Oslo, Norway). Leucosep tubes were supplied by Greiner (Frickenhausen, Germany). All other chemicals were of analytical grade.

### Patient and controls

All patients were treated for Dukes C colorectal cancer and participated in a protocol that had been designed to study 5-FU and DHFU pharmacokinetics. The protocol was approved by the Medical Ethics Review Committee of the Martini Hospital Groningen and written informed consent was obtained from all patients. All patients who entered this protocol were chemotherapy naive. Chemotherapy consisted of leucovorin 20 mg m^−2^ combined with 5-FU 425 mg m^−2^, both on 5 successive days, in a 28-day cycle. Blood sampling was carried out on the first day of the first chemotherapy cycle immediately following the 5-FU dose, administered as bolus intravenous injection over 2 min. Leucovorin was infused after the end of blood sampling. On the following 4 days, the same 5-FU dose was administered as short time infusion, after leucovorin administration. One patient experienced severe toxicity during the first chemotherapy cycle and, therefore, a screening on DPD deficiency was initiated. Data from six patients who participated in the same study protocol and who showed no signs of severe toxicity were randomly selected for reference pharmacokinetics, DPYD genotyping and DPD enzyme activity. These patients served as controls.

### Collection of blood samples

For pharmacokinetic sampling, a canule was placed in the arm of the patient contralateral from drug administration. Blood samples (5 ml) were collected in heparinised tubes just before, and 2, 5, 10, 20, 30, 45, 60, 80, 100, 120, 150 and 180 min postinjection. Collected samples were immediately placed on ice and subsequently centrifuged at 2500 *g* for 10 min. The plasma samples were analysed for 5-FU and DHFU concentrations by high-performance liquid chromatography (HPLC) on the day of collection. Blood samples for DPD analysis were collected 5 to 23 months after blood sampling for 5-FU pharmacokinetics, which corresponds to intervals ranging from 2 to 17 months after the last 5-FU dose. None of the patients received chemotherapy at that moment.

### Reversed phase HPLC analysis

5-Fluorouracil and DHFU concentrations were measured by HPLC analysis using a modification of the method described by Ackland *et al* (1997). Briefly, 100 μl chlorouracil internal standard solution (80 mg l^−1^ in water) was added to 1 ml plasma sample, and this mixture was vortexed and subsequently deproteinated with 50 μl of a 50% (w v^−1^) trichloracetic acid solution. After centrifugation at 8000 *g* for 2 min the supernatant was transferred into a 20 ml centrifuge tube and neutralised with 1 ml 1 M sodium acetate solution. Then 5 ml ethylacetate was added and the mixture was vortexed during 10 min. After separation of the organic and aqueous layers by centrifugation at 5000 *g* for 5 min, the ethylacetate layer was transferred into a 10 ml tube and evaporated under a stream of nitrogen at 25°C. The residue was dissolved in 100 μl ultrapure water and 20 μl was injected. 5-Fluorouracil and DHFU standards ranging from 0.5 to 20 mg l^−1^ were prepared in human plasma. The chromatographic system consisted of a Waters 616 pump equipped with a Waters 717+ autosampler. The separation of 5-FU and DHFU was accomplished by gradient elution at ambient temperature on a Phenomenex Prodigy ODS 3 column (I.D. 250×4.6 mm, 5 μm) equipped with a guard column (30×4.6 mm) of the same material. Mobile phase A consisted of 1.5 mM K_3_PO_4_ and 1% (v v^−1^) methanol (pH=6.0) and mobile phase B of 1.5 mM K_3_PO_4_ and 5% (v v^−1^) methanol (pH=6.0).

The gradient was programmed as follows: 100% A during 2 min; 100% A→100% B in 0.5 min; 100% B during 7 min; 100% B→100% A in 0.5 min; 100% A during 10 min. Detection was performed using a Waters 996 Photo Diode Array UV detector interfaced with a Millenium 2010 Chromatography Manager Workstation. Spectra were acquired in the 201–300 nm range. 5-FU was monitored at 266 nm and DHFU at 205 nm. The internal standard chlorouracil was monitored at both wavelengths.

### Pharmacokinetic analysis

The pharmacokinetic analyses were performed in the ADAPT II computer program (version 4.0; USC Los Angeles). The pharmacokinetic data of both the index patient and the six control patients were tested in eight different models. In each model the patient's data were fitted individually and for each data set the Akaike Information Criterion (AIC) was calculated. The model with the lowest summarised AIC value was selected as the better one (data not shown). The model used for calculating 5-Fluorouracil pharmacokinetics is a two-compartment model with Michaelis–Menten elimination from the first compartment and is described by two differential equations:


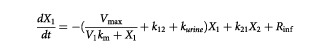



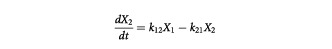


*X*_1_ and *X*_2_ indicate the amount of drug in each compartment, respectively. The *k*-values represent linear distribution- and elimination rate constants, and the *V*_max_ and *K*_m_ values represent Michaelis–Menten constants for non-linear elimination from the first compartment. *R*_inf_ represents the infusion rate of 5-FU.

The area under the curve of 5-FU and DHFU was calculated using the trapezoid rule. The average systemic clearance of 5-FU was calculated by dividing the administered dose by the area under the curve (AUC_0_→_3h_).

### Determination of dihydropyrimidine dehydrogenase activity

To investigate whether the 5-FU toxicity might have been caused by a partial deficiency of DPD, we determined the activity of DPD in peripheral blood mononuclear (PBM) cells. Therefore PBM cells were isolated from 15 ml EDTA anticoagulated blood and the activity of DPD was determined according previously described methods (Van Kuilenburg *et al*, 2000b). In brief, the sample was incubated in a reaction mixture containing 35 mM potassium phosphate pH 7.4, 1 mM dithiothreitol, 2.5 mM magnesium chloride, 250 μM NADPH and 25 μM [4-^14^C]thymine. After an appropriate incubation time, the reaction catalysed by DPD was terminated by adding 10% (v* *v^−1^) perchloric acid. The reaction mixture was centrifuged at 11 000* g* for 5 min to remove protein. The separation of radiolabelled thymine and the reaction products was performed by reversed phase HPLC. Protein concentrations were determined with a copper-reduction method using bicinchoninic acid, as described by Smith *et al* (1985).

### PCR amplification of coding exons

The DNA from the index and control patients was isolated from PBM cells as previously described (Van Kuilenburg *et al*, 1997). PCR amplification of exon 14 and flanking intronic regions was carried out according to Van Kuilenburg *et al* (2000a). PCR products were separated on 1% agarose gels, visualised with ethidium bromide and purified using a Qiaquick Gel Extraction kit and used for direct sequencing.

### Sequence analysis

Sequence analysis of the genomic fragment was carried out on an Applied Biosystems model 377 automated DNA sequencer using the Dye-Terminator method.

### Statistical analysis

Each value, measured in the index patient, was compared to the mean±2 s.d. range of the corresponding parameter in the control group. Values outside this range were considered abnormal (*P*<0.05). We did not match our control patients for age and gender.

## RESULTS

### Clinical evaluation

Patient characteristics from the index and control patients, as measured before 5-FU administration on the first day of the first chemotherapy cycle, are listed in Table 1

**Table 1:**
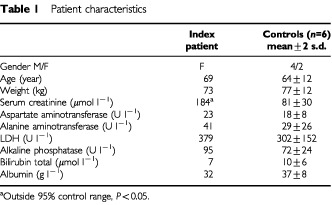
Patient characteristics

. The index patient is a 60-year-old white female who received adjuvant chemotherapy for Dukes C colon carcinoma. She was known with a chronic moderate renal function impairment as a result of a double-sided nephrolithotomy at age 40. The first two injections with a total dose of 800 mg 5-FU day^−1^ were tolerated well by the patient without complications. On the third day of chemotherapy she experienced nausea and cold shivers. The nausea was successfully treated with metoclopramide. The cold shivers remained on days 4 and 5. Twelve days after administration of the first 5-fluouracil injection, leukopenia (1.5×10^9^ leukocytes l^−1^) and thrombocytopenia (26×10^9^ platelets l^−1^) developed along with nausea, diarrhoea, stomatitis, fever and hair loss. The next day leukocytes and platelets decreased to 0.5×10^9 ^l^−1^ and 12×10^9 ^l^−1^ (both nadir values respectively). During this period the patient developed leukopenic fever (40°C) for which antibiotics were administered. Until day 20 the leucocytes and platelets remained low (1×10^9 ^l^−1^ and 13×10^9 ^l^−1^ respectively). During the subsequent week the clinical picture and hematological parameters gradually improved and normalised. On day 34 the patient was discharged from the hospital.

The toxicity observed in the six control patients was limited to mild nausea (*n*=4), vomiting (*n*=2) and CTC grade 1 stomatitis (*n*=1).

### Pharmacokinetic analysis

**Figure 2:**
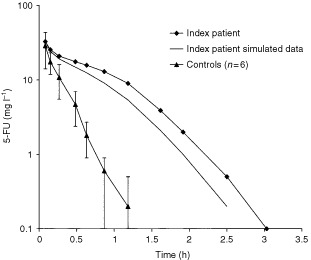
Pharmacokinetics of 5-FU. Shown are 5-FU plasma levels observed in a patient with a IVS14+1G>A mutation in the DPYD gene (solid diamond) and the 5-FU plasma levels resulting from simulation of a normal renal function in the same patient. 5-FU plasma levels from control patients are depicted as mean±s.d. (solid triangle; *n*=6).

The clearance of 5-FU was considerable slower in the index patient than in the six control patients. In all control patients the plasma level at *t*=90 min was below 0.1 mg l^−1^, whereas in the index patient the plasma level was still 3.8 mg l^−1^ at this time point (see Figure 2

). The AUC_0_→_3h_ in the patient suffering from toxicity was 24.1 mg h l^−1^ compared to 15.3 mg h l^−1^ as highest AUC_0_→_3h_ value in control patients. We calculated an average systemic clearance of only 520 ml min^−1^ *vs* 980–1780 ml min^−1^ in controls. The *V*_max_ value, calculated by pharmacokinetic modelling was 548 mg h^−1^, while the *V*_max_ values of control patients ranged from 984 to 1772 mg h^−1^ (see Table 2

**Table 2:**
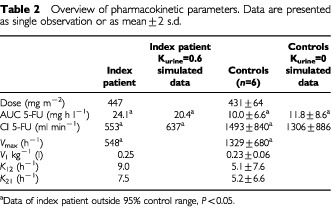
Overview of pharmacokinetic parameters. Data are presented as single observation or as mean±2 s.d.

). The pharmacokinetic data of the six control patients were comparable to data from literature (Port *et al*, 1991; Terret *et al*, 2000)

The effect of the impaired renal function of the index patient on 5-FU clearance was studied by pharmacokinetic modelling.

The excretion of 5-FU in urine was measured in five patients and k_urine_ was estimated 0.5±0.08 h^−1^. This k_urine_ value, individually normalised on calculated GFR, was used during subsequent modelling of other patient data. A normal renal function was simulated in the index patient by replacing the GFR related k_urine_ by k_urine_=0.6.

Renal function impairment appeared to have only a slight effect on 5-FU clearance. In the index patient, we estimated an additional 18% increase of the AUC due to the renal insufficiency upon a 108% higher AUC due to partial DPD deficiency.

Simulation of anuria in the control group (K_urine_=0), revealed a 16±4% increase of the 5-FU AUC.

### Activity of DPD in PBM cells

The activity of DPD in peripheral blood mononuclear (PBM) cells was lower in the patient experiencing severe toxicity (5.5 nmol mg^−1^ h^−1^) compared to the six control patients (8.0–11.7 nmol mg^−1^ h^−1^; mean 9.6) and comparable to obligate heterozygotes (Van Kuilenburg *et al*, 2000a)

### Genomic sequence analysis

Sequence analysis of the DPYD gene showed that the patient was heterozygous for a G→A point mutation in the invariant GT splice donor site (IVS14+1G>A), leading to the skipping of exon 14 directly upstream of the mutated splice donor site during DPD pre-mRNA splicing.

Sequence analysis of exon 14 of DPYD of the six control patients revealed no mutations.

## DISCUSSION

5-Fluorouracil remains the major drug in the treatment of advanced colorectal cancer. Dihydropyrimide dehydrogenase is the key metabolic enzyme in 5-FU degradation and since more than 80% of the dose is metabolised by this enzyme, DPD activity is one of the main factors determining drug exposure (Harris *et al*, 1990; Fleming *et al*, 1992a; Lu *et al*, 1993; Etienne *et al*, 1994). It is generally accepted that DPD activity in the liver is responsible for the majority of 5-FU catabolism (Ho *et al*, 1986; Fleming *et al*, 1992b), but PBM cells are often used as a surrogate for liver DPD activity, since these cells are better accessible (Harris *et al*, 1990; Fleming *et al*, 1992a; Lu *et al*, 1993; Etienne *et al*, 1994). Several groups have suggested that markedly diminished DPD activity in PBM cells is strongly related to the risk of developing severe 5-FU toxicity due to reduced 5-FU clearance (Lu *et al*, 1993; Etienne *et al*, 1994; Van Kuilenburg *et al*, 2000a). Although total DPD deficiency is rare in adults, about 2–3% of the population has a low PBM–DPD enzyme level and, thus, is at risk to develop severe toxicity when treated with 5-FU (Etienne *et al*, 1994; Lu *et al*, 1995; Chazal *et al*, 1996). In only few reports however, the effect of DPD-deficiency on 5-FU clearance has been objectively quantified. Diasio *et al* (1988) administered a test dose of 25 mg m^−2^ 5-FU to a patient with non-detectable DPD-activity in PBM cells and found a very low 5-FU clearance rate. This patient was probably homozygous for a mutant DPD allele, although the genetic cause was never elucidated. Stephan *et al* (1995) reported severe toxicity in a female patient after treatment comprising Leuvorin 500 mg m^−2^ as 2 h intravenous infusion plus 125 mg orally, followed by 5-FU 2 g m^−2^ as a 24 h continuous infusion. They found a 5-FU plasma level of 0.3 mg l^−1^ on day 15 after administration, which implies a dramatic overexposure to 5-FU. This patient could not have been homozygous deficient because the DPD activity in lymphocytes was within the normal range. The role of PBM–DPD activity as an indicator for 5-FU clearance is, however, questionable. Lu *et al* (1993) found a correlation between PBM–DPD activity and DPD activity measured in the liver, but others reported a weak, non-significant relationship (Chazal *et al*, 1996). Both Harris *et al* (1990) and Fleming *et al* (1992a) initially reported a correlation between PBM–DPD activity and 5-FU clearance but re-examination of this relationship in a larger set of patients revealed a markedly weakened correlation (Etienne *et al*, 1994). These data indicate that PBM-DPD activity is not a strong and reliable indicator of 5-FU clearance. To some extent, the variability in DPD activity might have been caused by the composition of the isolated PBM cells (Van Kuilenburg *et al*, 2000b). Another important factor is the timing of cell sampling in relation to 5-FU administration, since McLeod *et al* (1998) showed that 5-FU is able to inhibit DPD-activity.

In this paper, we report a combined pharmacokinetic and genetic analysis of the DPYD gene and demonstrate that a single G→A point mutation in the invariant splice donor site IVS14+1 of the DPYD gene has profound impact on the clearance of 5-FU. We also found a lower PBM–DPD activity in the index patient compared to six controls. The DPD activity was comparable to the activity observed in obligate heterozygotes (5.5±2.1 nmol mg^−1^ h^−1^, *n*=8) in previous work (Van Kuilenburg *et al*, 2000a), but the measured value also fits within the range for normal controls (10.0±3.4 nmol mg^−1^ h^−1^, range 3.4–18 nmol mg^−1^ h^−1^, *n*=22). This might indicate that not only obligate heterozygotes, but also low normal homozygotes are at risk for developing severe toxicity when treated with 5-FU. It is however as yet unclear whether the pharmacokinetic profile of 5-FU is identical in both groups.

We did not measure DPD activity and 5-FU pharmacokinetics on the same day. DPD activity was measured at least 2 months after completing chemotherapy and therefore might have been changed since the first chemotherapy cycle as a result of chemotherapy itself or disease state. Although 5-FU has a direct inhibitory effect on DPD activity, as was shown by McLeod *et al* (1998), we believe that it is not likely that this effect will continue until 2 months after the last dose. Furthermore, it has been shown that DPD activity is lower in breast-cancer patients compared to healthy persons (Lu *et al*, 1998), suggesting an effect of disease state on DPD activity. All our patients had Dukes C carcinoma and showed no signs of disease progression at the time of blood sampling for DPD. Therefore we considered the index patient and controls comparable regarding their disease state. Thus, despite delayed DPD sample collection, we believe our results to be representative for DPD activity during pharmacokinetic sampling.

The structural organization of the DPYD gene has recently been described. It is 150 kb in length and consists of 23 exons ranging in size from 69 to 1404 bp (Johnson *et al*, 1997). The G→A mutation changes an invariant GT splice donor site into AT which leads to skipping of a 165 bp exon immediately upstream of the mutated spice donor site during the splicing of DPD pre-mRNA. As a consequence, a 165 bp fragment encoding the amino acid residues 581–635 of the primary sequence of the DPD protein is lacking in the mature DPD mRNA, which results in an enzyme without catalytic activity (Wei *et al*, 1996; Van Kuilenburg *et al*, 1997). Analysis of the prevalence of the various mutations among cancer patients with partial DPD deficiency showed that the G→A mutation in the invariant splice donor site is the most common one (43%) (Van Kuilenburg *et al*, 2000a). The prevalence of this mutation in the normal Dutch population is 1.8% (Van Kuilenburg *et al*, 2001).

We believe that in our patient the IVS14+1G>A mutation explains the dramatic reduction in 5-FU clearance compared to controls. This is in line with the observation that at least 80% of the 5-FU dose is catabolised by DPD. Although our patient had a moderately impaired renal function, we analysed that the effect of renal function on 5-FU clearance is only limited. This was to be expected, as only about 10% of the 5-FU dose is normally excreted in urine (Heggie *et al*, 1987). It is important, however, to realise that in patients with reduced DPD capacity, the contribution of renal excretion to total clearance is relatively increased. As a consequence, the impact of renal insufficiency on the AUC is larger in DPD deficient than in normal patients. Thus, in the index patient, the impaired renal function might have contributed to development of more severe toxicity, additionally to that caused by DPD deficiency.

Development of rapid assays to detect mutations in the DPYD gene makes it possible to carry out a genetic screening prior to the start of chemotherapy containing 5-FU. However, it is important to identify those mutations that result in a defect DPD protein. So far, 19 molecular defects in the DPYD gene such as point mutations and deletions due to exon skipping have been reported, but not all mutations result in a DPD enzyme deficiency (Van Kuilenburg *et al*, 2000a). Incomplete correlation between DPD phenotype and genotype is clinically important and suggests that DPD polymorphisms are likely to be complex (Collie-Duguid *et al*, 2000).

Our results indicate that low DPD activity, due to the inactivation of one DPYD allele results in a strong reduction in 5-FU clearance, measured on the first chemotherapy day. Inhibition of the yet reduced DPD activity by 5-FU itself during subsequent days may lead to further reduction of 5-FU clearance and this may further add to the development of severe toxicity. In order to identify those mutations that result in reduced 5-FU clearance, monitoring of 5-FU plasma levels using a limited sampling strategy can be helpful in patient selection. This requires, however, rapid plasma level analysis, because results from the first 5-FU infusion must be available before the second dose is administered. Unfortunately, no rapid 5-FU (immuno-)assay is available yet, and therefore in most hospitals therapeutic drug monitoring of 5-FU is not yet feasible.

## References

[bib1] AcklandSPGargMBDunstanRH1997 Simultaneous determination of dihydrofluorouracil and 5-fluorouracil in plasma by high-performance liquid chromatography Anal Biochem 246 7985 9056186 10.1006/abio.1996.9943

[bib2] ChazalMEtienneMCReneeNBourgeonARichelmeHMilanoG1996 Link between dihydropyrimidine dehydrogenase activity in peripheral blood mononuclear cells and liver Clin Cancer Res 2 506510 9816197

[bib3] Collie-DuguidESEtienneMCMilanoGMcLeodHL2000 Known variant DPYD alleles do not explain DPD deficiency in cancer patients Pharmacogenetics 10 217223 10803677 10.1097/00008571-200004000-00002

[bib4] DiasioRBBeaversTLCarpenterJT1988 Familial deficiency of dihydropyrimine dehydrogenase. Biochemical basis for familial pyrimidinemia and severe 5-fluorouracil induced toxicity J Clin Invest 81 4751 3335642 10.1172/JCI113308 PMC442471

[bib5] EtienneMCLagrangeJLDassonvilleOFlemingRThyssAReneeNSchneiderMDemardFMilanoG1994 Population study of dihydropyrimidine dehydrogenase in cancer patients J Clin Oncol 12 22482253 7964939 10.1200/JCO.1994.12.11.2248

[bib6] FlemingRAMilanoGThyssAEtienneMCReneeNSchneiderMDemardF1992a Correlation between dihydropyrimidine dehydrogenase activity in peripheral mononuclear cells and systemic clearance of fluorouracil in cancer patients Cancer Res 52 28992902 1581906

[bib7] FlemingRAMilanoGAEtienneMCReneeNThyssASchneiderMDemardF1992b No effect of dose, hepatic function, or nutrional status on 5-FU clearance following continuous (5-day) 5-FU infusion Br J Cancer 66 668672 1419604 10.1038/bjc.1992.335 PMC1977433

[bib8] HarrisBESongRSoongSJDiasioRB1990 Relationship between dihydropyrimidine dehydrogenase activity and plasma 5-fluorouracil levels with evidence for circadian variation of enzyme activity and plasma drug levels in cancer patients receiving 5-fluorouracil by protracted continuous infusion Cancer Res 50 197201 2293556

[bib9] HeggieGDSommadosiJPCrossDSHusterWJDiasioRB1987 Clinical pharmacokinetics of 5-fluorouracil and its metabolites in plasma, urine and bile Cancer Res 47 22032206 3829006

[bib10] HoDHTownsendLLunaMBodeyGP1986 Distribution and inhibition of dihydrouracil dehydrogenase activities in human tissues using 5-fluorouracil as substrate Anticancer Res 6 781784 3752956

[bib11] JohnsonMRWangKTillmannsSAlbinNDiasioRB1997 Structural organization of the human dihydropyrimidine dehydrogenase gene. Cancer Res 57 16601663 9135003

[bib12] LuZZhangRDiasioRB1993 Dihydropyrimine dehydrogenase activity in human peripheral blood mononuclear cells and liver: population characteristics, newly identified patients, and clinical implication in 5-fluorouracil chemotherapy Cancer Res 53 54335438 8221682

[bib13] LuZZhangRDiasioRB1995 Population characteristics of hepatic dihydropyrimidine dehydrogenase activity, a key metabolic enzyme in 5-fluorouracil chemotherapy Clin Pharmacol Ther 58 512522 7586945 10.1016/0009-9236(95)90171-X

[bib14] LuZZhangRCarpenterJTDiasioRB1998 Decreased dihydropyrimine dehydrogenase activity in a population of patients with breast cancer: implication for 5-fluorouracil-based chemotherapy Clin Cancer Res 4 325329 9516918

[bib15] McLeodHLSluddenJHardySCLockREHawksworthGMCassidyJ1998 Autoregulation of 5-fluorouracil metabolism Eur J Cancer 34 16231627 9893640 10.1016/s0959-8049(98)00175-0

[bib16] PinedoHMPetersGF1988 Fluorouracil: biochemistry and pharmacology J Clin Oncol 6 16531664 3049954 10.1200/JCO.1988.6.10.1653

[bib17] PortREEdlerLHerrmannRFeldmannU1991 Pharmacokinetics of 5-fluorouracil after short systemic infusion: plasma level at the end of the distribution phase as an indicator of the total area under the plasma concentration – time curve Ther Drug Mon 13 96102 2053130

[bib18] SmithPKKrohnRIHermansonGTMalliaAKGartnerFHProvenzanoMDFujimotoEKGoekeNMOlsonBJKlenkDC1985 Measurement of protein using bicinchoninic acid Anal Biochem 150 7685 3843705 10.1016/0003-2697(85)90442-7

[bib19] StephanFEtienneMCWallaysCMilanoGClergueF1995 Depressed hepatic dihydropyrimidine dehydrogenase activity and fluorouracil related toxicities Am J Med 99 685688 7503095 10.1016/s0002-9343(99)80259-9

[bib20] TerretCErdociainEGuimbaudRBoisdron-CelleMMcLeodHLFety-DeporteRLafondTGamelinEBugatRCanalPChatelutE2000 Dose and time dependencies of 5-fluorouracil pharmacokinetics Clin Pharmacol Ther 68 270279 11014408 10.1067/mcp.2000.109352

[bib21] Van KuilenburgABPVrekenPBeexLVMeinsmaRVan LentheHDeAbreuRAVan GennipAH1997 Heterozygosity for a point mutation in an invariant splice donor site of dihydropyrimine dehydrogenase and severe 5-fluorouracil related toxicity Eur J Cancer 33 22582264 9470816 10.1016/s0959-8049(97)00261-x

[bib22] Van KuilenburgABPHaasjesJRichelDJZoetekouwLVan LentheHWaterhamHRDe AbreuRAMaringJGVrekenPVan GennipAH2000a Clinical implications of dihydropyrimidine dehydrogenase (DPD) deficiency in patients with severe 5-fluorouracil associated toxicity. Identification of new mutations in the DPD gene Clin Cancer Res 6 47054712 11156223

[bib23] Van KuilenburgABPVan LentheHTrompAVeldmanPCVan GennipAH2000b Pitfalls in the diagnosis of patients with partial dihydropyrimidine dehydrogenase deficiency Clin Chem 46 917 10620566

[bib24] Van KuilenburgABPMullerEWHaasjesJMeinsmaRZoetekouwLWaterhamHRBaasFRichelDJVan GennipAH2001 Lethal outcome of a patient with complete dihydropyrimidine dehydrogenase (DPD) deficiency after administration of 5-fluorouracil: frequency of the common IVS14+1G>A mutation causing DPD deficiency Clin Cancer Res 7 11491153 11350878

[bib25] WeiXMcLeodHLMcMurroughJGonzalezFJFernandez-SalgueroP1996 Molecular basis of the human dihydropyrimidine dehydrogenase deficiency and 5-fluorouracil toxicity J Clin Invest 98 610615 8698850 10.1172/JCI118830 PMC507468

